# Boron Phosphide: A Comprehensive Overview of Structures, Properties, Synthesis, and Functional Applications

**DOI:** 10.3390/nano15090654

**Published:** 2025-04-25

**Authors:** Qilong Wu, Jiamin Wu, Maoping Xu, Yi Liu, Qian Tian, Chuang Hou, Guoan Tai

**Affiliations:** State Key Laboratory of Mechanics and Control for Aerospace Structures, Laboratory of Intelligent Nano Materials and Devices of Ministry of Education, College of Aerospace Engineering, Nanjing University of Aeronautics and Astronautics, Nanjing 210016, China; sz2201086@nuaa.edu.cn (Q.W.); wujiamin@nuaa.edu.cn (J.W.); mpx_ins@nuaa.edu.cn (M.X.); liuyi061@nuaa.edu.cn (Y.L.); sx2201054@nuaa.edu.cn (Q.T.)

**Keywords:** boron phosphide, boron, chemical vapor deposition, photodetectors, sensors, thermal management, energy

## Abstract

Boron phosphide (BP), an emerging III–V semiconductor, has garnered significant interest because of its exceptional structural stability, wide bandgap, high thermal conductivity, and tunable electronic properties. This review provides a comprehensive analysis of BP, commencing with its distinctive structural characteristics and proceeding with a detailed examination of its exceptional physicochemical properties. Recent progress in BP synthesis is critically examined, with a focus on key fabrication strategies such as chemical vapor deposition, high-pressure co-crystal melting, and molten salt methods. These approaches have enabled the controlled growth of high-quality BP nanostructures, including bulk crystals, nanoparticles, nanowires, and thin films. Furthermore, the review highlights the broad application spectrum of BP, spanning photodetectors, sensors, thermal management, energy conversion, and storage. Despite these advances, precise control over the growth, morphology, and phase purity of BP’s low-dimensional structures remains a critical challenge. Addressing these limitations requires innovative strategies in defect engineering, heterostructure design, and scalable manufacturing techniques. This review concludes by outlining future research directions that are essential for unlocking BP’s potential in next-generation electronics, sustainable energy technologies, and multifunctional materials.

## 1. Introduction

Boron (B) and phosphorus (P), respectively, belong to groups III and V of the periodic table and exhibit distinct electronic configurations and bonding characteristics that underpin their diverse material behaviors. Boron, with only three valence electrons, is markedly electron deficient [1,2]. Its atoms typically adopt sp^2^ or sp^3^ hybridization to form multicenter bonds, such as three-center B–B–B bonds, which are conducive to the synthesis of various low-dimensional materials [3,4,5,6,7,8], including hexagonal boron nitride (*h*-BN) and two-dimensional (2D) borophene. These bonding modes impart exceptional mechanical strength and electrical performance to boron-based materials [9]. In contrast, phosphorus is characterized by its variable valence states (ranging from −3 to +5) and significant lone pair effects, leading to diverse allotropes such as white, red, and black phosphorus, whose semiconducting properties can be tuned via interlayer van der Waals interactions [10,11,12]. Due to their unique bonding characteristics and structural versatility, boron-based and phosphorus-based materials have attracted significant research interest [3,4,5,6,7,8,9,10,11,12]. In particular, the combination of boron and phosphorus results in new chemical bonding characteristics and potential functionalities. Theoretical studies indicate that the strongly polar B-P bond stabilizes sp^3^-hybridized networks while inducing charge redistribution, which in turn affects charge transport behavior [13,14]. This interaction between bonding characteristics provides a solid theoretical foundation for the development of boron phosphide (BP).

**Figure 1:**
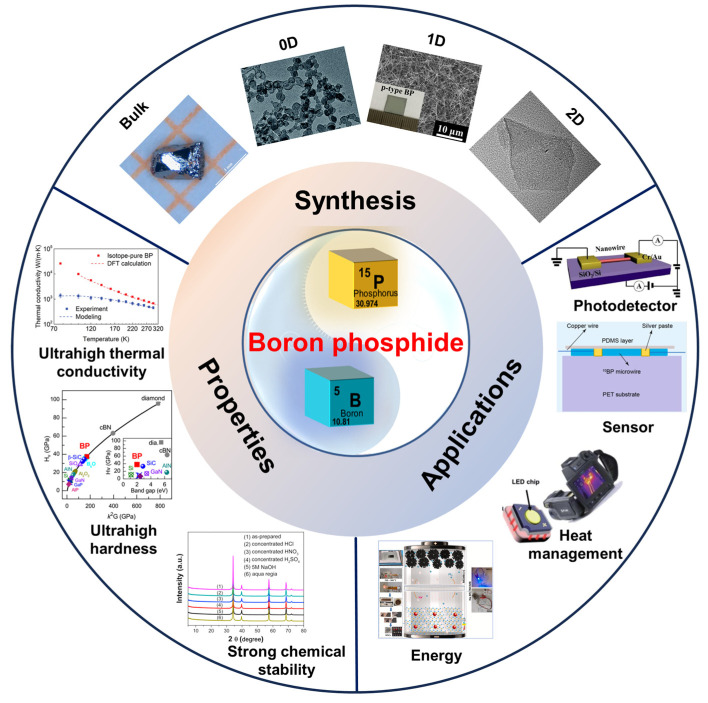
Properties, synthesis, and applications of boron phosphide [20,29,30,42,44,47,48,49,50,51]. Properties: Ultrahigh thermal conductivity. Reprinted with permission from [20], Copyright 2017, American Chemical Society. Ultrahigh hardness. Reprinted with permission from [30], Copyright 2020, American Physical Society. Strong chemical stability. Reprinted with permission from [29], Copyright 2016, Elsevier Ltd. Bulk boron phosphide. Reprinted with permission from [42], Copyright 2021, Elsevier Ltd. 0D boron phosphide. Reprinted with permission from [48], Copyright 2019, Royal Society of Chemistry. 1D boron phosphide. Reprinted with permission from [44], Copyright 2018, American Chemical Society. 2D boron phosphide. Reprinted with permission from [49], Copyright 2020, Elsevier B.V. Photodetector. Reprinted with permission from [44], Copyright 2018, American Chemical Society. Sensor. Reprinted with permission from [50], Copyright 2022, American Chemical Society. Heat management. Reprinted with permission from [47], Copyright 2021, Royal Society of Chemistry. Energy. Reprinted with permission from [51], Copyright 2023, Elsevier B.V.

BP, a III-V compound semiconductor, has garnered significant attention because of its exceptional structural and electronic properties [15,16,17,18,19,20,21,22,23,24,25,26]. Although numerous BP structures have been reported, attributed to the complex bonding nature of boron and phosphorus, research has primarily focused on two key polymorphs: cubic and hexagonal BP (*h*-BP). In cubic BP (*c*-BP), boron and phosphorus atoms adopt tetrahedral coordination to construct a stable three-dimensional (3D) covalent network characterized by a lattice constant of approximately 0.454 nm [27] and an indirect band gap near 2.1 eV [28,29]. Furthermore, *c*-BP exhibits a high load-invariant hardness of up to 38 GPa [30] and maintains thermal conductivity around 500 W·m^−1^·K^−1^ [31], as well as keeps high-temperature stability over 1800 K (Figure 1). In contrast, *h*-BP features a layered stacking arrangement in which in-plane B-P hexagonal rings resemble those found in graphene [32], while pronounced polarization effects in the interlayer region yield anisotropic carrier mobility with theoretical values exceeding 1000 cm^2^·V^−1^·s^−1^. Moreover, through doping (e.g., with Si or O) or the construction of heterostructures, its electronic structure can be precisely tuned from *n*-type to *p*-type, providing significant advantages over other medium- to narrow-band-gap semiconductors [32,33,34,35,36,37,38,39,40]. However, the synthesis of high-quality BP remains a significant challenge because of the high melting point of boron and the volatility of phosphorus, necessitating precise control over fabrication conditions [41].

Recent advancements in synthesis techniques (Figure 1), including high-pressure co-crystal melting [30,42], chemical vapor deposition (CVD) [43,44], and molten salt-assisted routes [45,46,47], have significantly expanded the possibilities for fabricating BP structures with well-defined morphology, high phase purity, and tunable defect concentrations [42,44,48,49]. These breakthroughs have not only enhanced the crystallinity and electronic properties of BP but also enabled precise control over its dimensionality, opening new avenues for device integration. In particular, BP nanowires and thin films have garnered substantial interest because of their outstanding charge carrier mobility, mechanical flexibility, and optical tunability. These low-dimensional structures have exhibited remarkable performance in field-effect transistors (FETs) [44], demonstrating promising electrical transport characteristics that make them viable candidates for high-speed and low-power electronic devices (Figure 1). Additionally, their strong light absorption and fast carrier dynamics have been utilized in high-sensitivity photodetectors [44,50], offering rapid response times and broadband detection capabilities. Notably, the high theoretical capacity and rapid ion transport of BP have been leveraged in energy storage systems, including supercapacitor [51] (Figure 1). The inherent flexibility of BP thin films further enables their application in wearable and flexible sensing devices [52], where their excellent mechanical stability and high surface-to-volume ratio contribute to enhanced sensitivity and durability under repeated mechanical deformation. Beyond electronic and optoelectronic applications, BP nanoparticles [29,53,54] have emerged as promising materials for electrocatalysis, particularly in photocatalytic hydrogen production and CO_2_ reduction reactions (CO_2_RR). Their unique electronic structure and tunable active sites facilitate efficient charge transfer and catalytic activity, making them competitive alternatives to conventional noble-metal-based catalysts. Moreover, the high thermal conductivity and structural robustness of BP have positioned it as a compelling candidate for energy storage and thermal management applications, where it can serve as an efficient heat dissipation material or anode component in next-generation battery technologies (Figure 1).

To ensure a comprehensive and balanced overview of recent advances in boron phosphide, we conducted a systematic literature survey using databases such as Web of Science and Google Scholar. The search, limited to publications prior to 2025, used keywords including “boron phosphide”, “BP synthesis”, “thermal conductivity and boron phosphide”, “III–V semiconductors”, and “energy storage”. Only peer-reviewed journal articles and English-language reviews were included. We prioritized studies providing detailed experimental procedures, theoretical insights, or application-relevant performance evaluations and excluded those lacking methodological clarity or scientific rigor. This approach ensures the accuracy, relevance, and completeness of the literature reviewed in this article.

Despite these advancements, few reviews have devoted attention to covering the experimental developments of BP for application fields, especially sensors, thermal management, and energy. Based on the current literature, this review represents the first comprehensive summary and discussion of recent advancements in BP research, providing a systematic analysis of its structural characteristics, synthesis strategies, and emerging applications. By consolidating the latest findings and addressing existing challenges, we aim to highlight the future prospects of BP as a multifunctional material, guiding further research toward its widespread technological integration.

## 2. Structures of Boron Phosphide

**Figure 2:**
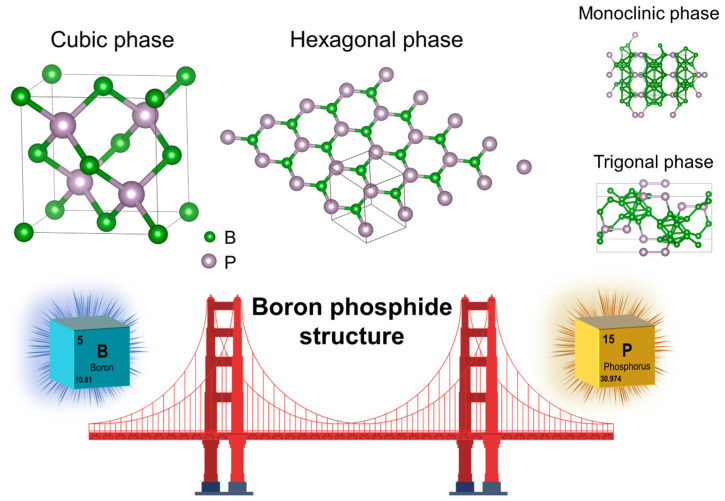
Different structures of boron phosphide.

Figure 2 presents the observed typical crystal structures of BP, encompassing cubic [15], hexagonal [32], trigonal [55], and monoclinic phases [55]. In nature, boron phosphide predominantly crystallizes in the zinc-blende phase (space group F4$\overset{-}{3}$m), known as cubic boron phosphide (*c*-BP), with a lattice parameter of a = 4.538 Å. The first successful synthesis of bulk *c*-BP was first reported as early as 1957 [15]. In recent years, extensive research efforts have enabled the fabrication of various micro- and nanostructures, including BP nanoparticles [29,54], nanowires [44,50,52], and thin films [43,49]. *c*-BP exhibits exceptional physicochemical properties, including high thermal conductivity, remarkable hardness, strong chemical stability, and an indirect bandgap [20,29,30]. These properties endow *c*-BP with significant potential for applications in thermal management systems, high-temperature electronics, neutron detectors, radiation shielding coatings, and optoelectronic devices.

Apart from the cubic phase, *h*-BP represents another crucial crystalline form of BP, crystallizing in the P6_3_mc space group with computed bond lengths of 1.858 Å and lattice parameters a = 3.19 Å and c = 5.29 Å. Theoretical investigations have predicted the existence of nanosheets and nanoribbons based on this structure [32,39,56]. Notably, Alejandro et al. [32] proposed that *h*-BP nanosheets exhibit electronic properties analogous to those of graphene and *h*-BN, with a bandgap intermediate between the two, owing to the electronegativity disparity between boron and phosphorus. Despite its advantageous electronic properties and application prospects, the synthesis of *h*-BP remains highly challenging because of the stringent growth conditions required, along with its pronounced sensitivity to impurities and defects.

Beyond the cubic and hexagonal phases, boron phosphide also exists in a trigonal structure, such as rhombohedral B_12_P_2_ [26,42,57]. This phase crystallizes in the R$\overset{-}{3}$m space group with lattice parameters a = 5.985 Å and c = 11.842 Å. Its 3D network is composed of B_12_ icosahedra interconnected by P-P chains, imparting enhanced thermal stability [57]. Experimentally, B_12_P_2_ can be synthesized via the thermal decomposition of cubic BP at elevated temperatures (~1120 °C), accompanied by phosphorus gas evolution [26]. At 1250 °C, temperature-dependent coalescence and coarsening of B_12_P_2_ grains are observed, leading to a significant increase in particle size from 30 nm to 500 nm. Remarkably, B_12_P_2_ exhibits exceptional thermal stability and superhigh hardness (Hv = 35–37 GPa), as confirmed by previous studies [57]. In addition to rhombohedral B_12_P_2_, a monoclinic BP phase has been theoretically proposed, crystallizing in the C1m1 space group with lattice parameters a = 8.68 Å, b = 5.92 Å, and c = 10.20 Å. However, its experimental realization remains elusive. Given that current research primarily focuses on the cubic and hexagonal phases, this review will center on their synthesis, properties, and potential applications.

## 3. Properties of Boron Phosphide

BP features robust covalent bonding between boron and phosphorus atoms, resulting in a dense crystalline structure. This unique bonding imparts several remarkable properties to BP, including exceptional hardness, high thermal stability, and tunable electronic behaviors [20,29,30,40,58]. The mechanical and thermal robustness of BP is primarily attributed to the nature of its crystal structure, while its electrical and chemical properties are influenced by its semiconducting nature and structural integrity. In the following sections, we explore these properties in detail, focusing on the mechanical, thermodynamic, electrical, and chemical characteristics of BP.

### 3.1. Mechanical Properties

**Figure 3:**
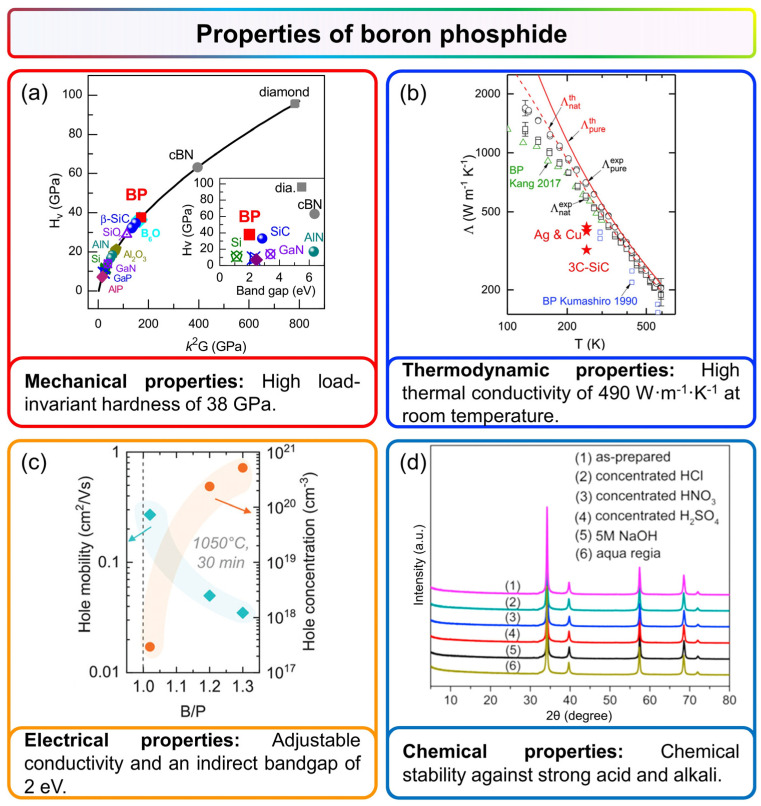
Properties of boron phosphide [29,30,40,58]. (**a**) Mechanical stability: The Vickers hardness (Hv) as a function of *k^2^G* (where *k* = *G*/*B*) for various semiconductors is presented. The inset illustrates the correlation between hardness and band gap for typical refractory semiconductors. Reprinted with permission from [30], Copyright 2020, American Physical Society. (**b**) Thermal conductivity: The thermal conductivity of BP and ^11^BP is shown over a temperature range of 120–600 K. Reprinted with permission from [58], Copyright 2018, Wiley-VCH. (**c**) Electrical properties: The hole mobility and concentration are plotted as a function of initial composition for three films annealed at 1050 °C. Reprinted with permission from [40], Copyright 2022, Wiley-VCH. (**d**) Chemical stability: XRD patterns of BP are displayed before and after heat treatment, following exposure to concentrated HCl, concentrated H_2_SO_4_, concentrated HNO_3_, aqua regia, and 5 M NaOH for 20 h. Reprinted with permission from [29], Copyright 2016, Elsevier Ltd.

In 1957, Popper et al. [15] first synthesized *c*-BP using a high-temperature vacuum sealing method. X-ray diffraction (XRD) revealed traces of tungsten carbide, implying that *c*-BP possesses an exceptional abrasiveness and may even exceed silicon carbide (SiC) in hardness. Since then, research on the mechanical properties of BP has advanced significantly. Recent studies have further quantified its hardness. Solozhenko et al. [59] reported that the Vickers hardness of BP reaches up to 30 GPa. Subsequent experiments by Gui et al. [30] demonstrated that bulk BP exhibits pronounced strain hardening, with a load-invariant hardness of approximately 38 GPa, which is close to the 40 GPa benchmark typically used to define superhard materials (Figure 3a). Feng et al. [60] further confirmed the phase stability of *c*-BP under static pressures up to 36 GPa, with full recovery upon decompression. More recently, Lu et al. [50,52] synthesized isotopically enriched ^10^BP microwires exhibiting hardness values as high as 41 GPa, along with remarkable elastic deformation capabilities. For *h*-BP, theoretical investigations by Sahin et al. [61] and Cakir et al. [39] have predicted that a monolayer with hexagonal symmetry is mechanically stable, with elastic constants and phonon spectra suggesting a performance intermediate between that of MoS_2_ and *h*-BN.

### 3.2. Thermodynamic Properties

Cubic BP exhibits a nearly symmetric electron charge distribution and strong covalent B-P bonding, leading to low ionicity and exceptional thermal robustness. Liang et al. [42] systematically assessed the thermal stability of large BP crystals over a broad temperature range (30–1200 °C) using thermal gravimetry/differential thermal analysis (TG-DTA), reporting a negligible weight loss of less than 0.2% (Figure 3b), highlighting its remarkable thermal resilience.

Beyond its intrinsic thermal stability, *c*-BP exhibits remarkably high thermal conductivity. Theoretical calculations by Lindsay et al. [31] predicted that *c*-BP could achieve an ultrahigh thermal conductivity of up to 580 W·m^−1^·K^−1^. In 2017, Zheng et al. [58] employed time-domain thermoreflectance (TDTR) to experimentally determine a thermal conductivity of ~490 W·m^−1^·K^−1^ at room temperature, which further increased to 540 W·m^−1^·K^−1^ in isotopically enriched ^11^BP. These values not only closely align with theoretical predictions but also surpass those of conventional high-thermal-conductivity materials such as Ag, Cu, and SiC, positioning BP among the most thermally conductive materials, second only to select compounds such as cubic boron nitride (*c*-BN), boron arsenide (BAs) and diamond [62,63,64,65,66,67,68,69,70,71,72]. Thus, BP can be selected as an effective thermal filler in composite materials. Hu et al. [46] demonstrated that incorporating BP nanoparticles significantly improves both the thermal stability and thermal conductivity of epoxy resin-based composites (BP-BN/EP), expanding their potential for high-performance thermal management applications.

### 3.3. Electrical Properties

Early studies on the electrical properties of BP date back to the 1970s [73,74,75], when Nishinaga et al. identified BP as an *n*-type semiconductor through thermoelectric potential measurements, reporting a resistivity of 5 × 10^−3^ Ω·cm. Around the same period, Shohno et al. [74] successfully synthesized BP single crystal layers on silicon substrates using vapor-phase epitaxy, demonstrating that the conductivity type of BP could be modulated by adjusting the substrate temperature and gas source ratio. Their studies revealed an electron concentration of 4 × 10^18^ cm^−3^ for *n*-type BP, with an electron mobility of approximately 140 cm^2^·V^−1^·s^−1^ at room temperature. Notably, *p*-type BP exhibited a significantly higher hole concentration of 5 × 10^19^ cm^−3^ and an exceptionally high hole mobility of 350 cm^2^·V^−1^·s^−1^, underscoring its potential for high-performance electronic applications.

With the advancement of theoretical modeling and experimental techniques, the electronic properties of BP have been further elucidated in recent years. In 2016, Shi et al. [29] confirmed BP’s *n*-type nature and an indirect bandgap of approximately 2 eV by integrating first-principles calculations with photoluminescence (PL) spectroscopy. Based on this, Varley et al. [76] employed G_0_W_0_ quasiparticle corrections to regulatory the bandgap estimation to 2 eV, consistent with experimental findings, while also determining a hole effective mass of 0.35m_0_. Further studies by Crovetto et al. [40] showed that by controlling carbon and silicon doping, the conductivity properties of BP are adjustable, achieving an enhanced hole concentration of up to 5 × 10^20^ cm^−3^ (Figure 3c). Therefore, the applicability of BP in optoelectronic and semiconductor devices has been further expanded.

Beyond the conventional cubic phase, theoretical studies have predicted intriguing electronic behaviors in low-dimensional BP structures. Using first-principles DFT combined with the non-equilibrium Green’s function method, Dong et al. [56] predicted that zigzag-edged *h*-BP nanoribbons behave as non-magnetic direct-bandgap semiconductors with a bandgap of approximately 1 eV, which decreases as the nanoribbon width increases. Interestingly, the application of a transverse electric field can induce a transition from a non-magnetic direct-bandgap semiconductor to a non-magnetic indirect-bandgap semiconductor, a ferromagnetic semiconductor, or even a semimetal, highlighting BP’s potential for tunable electronic and spintronic applications. Furthermore, Cakir et al. [39] predicted that monolayer *h*-BP possesses a direct bandgap of 0.91 eV at the *K*-point, further expanding its prospects for next-generation optoelectronic and quantum devices.

### 3.4. Chemical Stability

BP exhibits remarkable chemical stability, primarily attributed to the strong covalent bonding between boron and phosphorus atoms and its dense crystalline structure [77,78,79,80]. This exceptional stability was first documented in 1960 by Williams et al. [77], who demonstrated BP’s resistance to various corrosive environments. Their experiments showed that BP remains unetched in boiling concentrated mineral acids, including hydrochloric acid (HCl), sulfuric acid (H_2_SO_4_), and nitric acid (HNO_3_), as well as in aqua regia, even under prolonged reflux conditions. Notably, no significant weight loss or structural degradation was observed, underscoring BP’s superior chemical inertness compared with many other III-V semiconductors.

More recent investigations have further corroborated BP’s resilience under extreme chemical conditions. Shi et al. [29] conducted systematic stability tests, confirming that BP not only withstands concentrated HCl but also exhibits strong resistance to alkaline environments. Their study revealed that BP remains structurally and chemically intact even after prolonged exposure to 5 M NaOH at elevated temperatures for over 20 h (Figure 3d). Despite these harsh conditions, no discernible changes in BP’s crystal structure or surface composition were detected, reaffirming its robustness against both acidic and alkaline degradation.

Table 1 shows that BP uniquely combines high thermal conductivity (~540 W/m·K), exceptional mechanical hardness (~38 GPa), and balanced electron and hole mobilities (approximately 500 cm^2^/V·s), as well as a moderate indirect bandgap (~2.0 eV) [30,58]. This unique combination ensures excellent heat dissipation and ultrahigh mechanical robustness, which are rarely found together in conventional III-V or silicon-based semiconductors [81,82,83,84,85,86]. Such a balanced performance makes BP particularly attractive for applications requiring both high power handling and stable electronic properties.

In addition, boron phosphide also exhibits exceptional chemical stability in both highly acidic and alkaline environments, standing in stark contrast to conventional III-V semiconductors such as GaAs and InP. While materials such as GaAs undergo significant surface oxidation (e.g., forming Ga_2_O_3_/As_2_O_3_ layers in acidic conditions) and InP suffers from hydrolytic degradation in alkaline media due to weak In-P bond resilience, BP maintains structural integrity and surface passivity across extreme pH ranges (pH 0–14). Such unparalleled stability positions BP as a prime candidate for applications requiring harsh-environment durability, including photoelectrochemical water splitting, acid/alkaline-resistant optoelectronics, and catalytic systems.

## 4. Synthesis of Boron Phosphide

Given the exceptional physicochemical properties of BP, the synthesis of high-quality BP remains a crucial goal in this field. Research on *h*-BP has been primarily theoretical; therefore, this section focuses on the synthesis of cubic BP and its nanostructures. Since Popper and Ingles [15] first successfully synthesized bulk BP using a high-temperature vacuum-sealing technique in 1957, research on *c*-BP synthesis has continued unabated. Over the years, new fabrication techniques have emerged, leading to the development of diverse BP micro- and nanostructures.

### 4.1. Bulk Boron Phosphide

**Figure 4:**
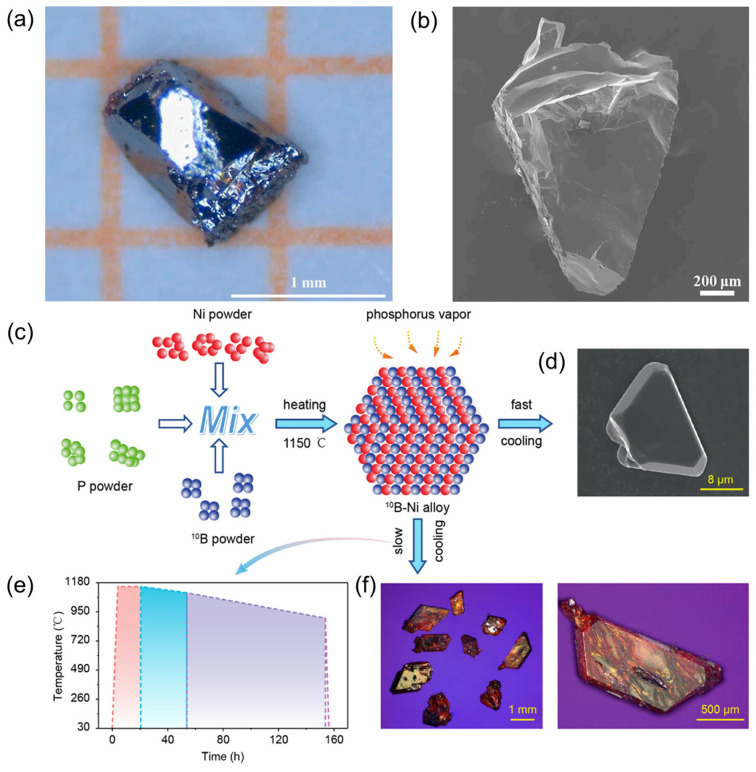
Synthesis of bulk boron phosphide [42,45]. (**a**) Optical image of a BP single crystal synthesized at 5.0 GPa and 3000 °C with a 10 min dwell time and a cooling rate of 10 °C/min. (**b**) SEM image of a BP single crystal, showcasing its microstructure. Reprinted with permission from [42], Copyright 2021, Elsevier Ltd. (**c**) Schematic illustration of the growth process for single-isotope ^10^BP. (**d**) SEM image of BP crystals obtained after rapid cooling. (**e**) Programmed cooling curve utilized for the growth of a millimeter-scale single crystal. (**f**) Optical image of a millimeter-scale ^10^BP after slow cooling. Reprinted with permission from [45], Copyright 2023, American Chemical Society.

Early studies primarily focused on methods for growing bulk BP crystals [15], but recent advancements have aimed at refining synthesis techniques to enhance crystal quality [42]. In 2021, Liang et al. [42] successfully synthesized millimeter-scale single-crystalline BP using a eutectic melting method under extreme conditions of 5.0 GPa and 3000 °C (Figure 4a). Scanning electron microscopy (SEM) images revealed well-defined crystal morphology (Figure 4b). While this approach effectively yields high-quality BP crystals, its requirement for exceptionally high temperatures and pressures poses significant challenges for scalability and broader practical applications.

To address these limitations, Zheng’s group [45] employed a gas–liquid–solid method using a high-temperature molten salt approach. By adjusting the ratio of isotopically enriched boron precursors, they synthesized ^nat^BP, ^10^BP, and ^11^BP. Importantly, the addition of nickel powder as a catalyst allowed for a significant reduction in the synthesis temperature of bulk BP. As shown in Figure 4c, a single-temperature CVD furnace was employed, with boron–nickel mixed powder and red phosphorus powder placed at opposite ends of a quartz tube. Under vacuum conditions and upon heating to 1150 °C, red phosphorus sublimed while the boron–nickel mixture melted, and nucleation ensued over approximately 1000 min at 1150 °C, followed by natural cooling to room temperature. This process yielded ^10^BP crystals ranging from a few to several tens of micrometers in size (Figure 4d). Furthermore, Zheng et al. also explored controlled cooling processes to promote crystal growth further. Cooling rates of 1.5 °C/h and 2 °C/h down to 1000 °C and 900 °C, respectively, enhanced nucleation and growth kinetics (Figure 4e), whereas rapid quenching at 350 °C/h produced large single crystals of ^10^BP (Figure 4f).

### 4.2. Boron Phosphide Nanoparticles

Nanoparticles, owing to their small dimensions and high specific surface area, offer a wealth of active sites beneficial for catalytic processes. Due to their exceptional thermal and chemical stability [29,42,77,78,79,80], BP nanoparticles can become a prime candidate for photocatalytic applications, particularly in harsh acid-base environments. Thus, the development of efficient and scalable synthesis methods for BP nanoparticles is crucial to fully harnessing their potential in photocatalytic applications, especially in demanding environments.

Shi et al. [29] reported the synthesis of a high-purity BP powder through a solid-phase reaction method, effectively avoiding the complexity of traditional high-pressure preparation methods. Moreover, the synthesized BP exhibited high charge mobility and low resistivity. After ball-milling treatment, the particle size could be reduced to a submicron level, and the significantly increased specific surface area further enhanced the photogenerated carrier separation efficiency and photocatalytic hydrogen production activity. Building on this, Hu et al. [46] explored a molten salt method for preparing BP nanoparticles, which significantly simplified the preparation process. This study employed three systems: single salt (NaCl), double salt (NaCl:KCl), and triple salt (NaCl:KCl:LiCl). The results showed that the triple salt method significantly improved the yield to 74%. Subsequently, Feng et al. [60] used a high-temperature vacuum-sealed solid-phase reaction method to further increase the yield of BP nanoparticles to 90–95%. These prepared BP particles demonstrated excellent structural stability under high pressure, withstanding up to 36 GPa without phase transition and exhibiting high reversible recovery capability. Although these techniques yield BP particles with sizes spanning hundreds of nanometers to micrometers, additional ball-milling is frequently required to achieve the ultrafine dimensions necessary for effective catalytic applications.

**Figure 5:**
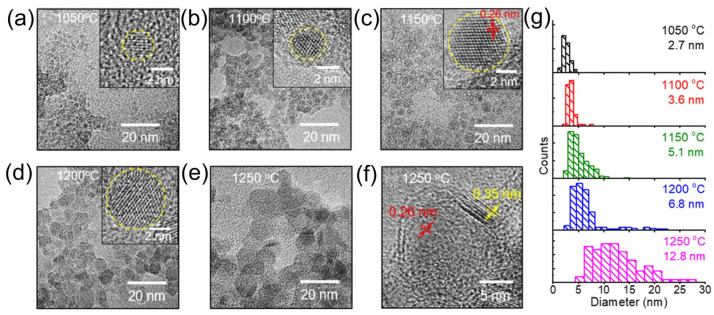
TEM images of BP nanocrystals at various temperatures [54]. (**a**–**e**) TEM images of BP NCs synthesized at temperatures ranging from 1050 °C to 1250 °C. The insets in (**a**–**d**) provide high-resolution TEM images, revealing detailed structural features. (**f**) High-resolution TEM image of BP NCs grown at 1250 °C, highlighting their crystallinity and morphology. (**g**) Particle size distribution histogram derived from TEM images, illustrating the size uniformity of the synthesized NCs. Reprinted with permission from [54], Copyright 2019, American Chemical Society.

To fully exploit BP’s intrinsic properties for photocatalysis and related applications, Sugimoto et al. [54] introduced an innovative method for synthesizing ultrafine BP nanocrystals (NCs). In this approach, radio-frequency sputtering was employed to deposit a borophosphosilicate glass (BPSG) film onto a thin stainless-steel substrate. Subsequent annealing in a vacuum environment at temperatures between 1050 and 1250 °C promoted the in situ growth of BP NCs within the BPSG matrix. Notably, the annealing temperature exerted precise control over the NC size (Figure 5a–f). High-resolution TEM images (HRTEM) (Figure 5a–d) revealed lattice spacings of approximately 0.26 nm, corresponding to the (111) planes of *c*-BP, while the size distribution analysis (Figure 5g) indicated that the average diameter of BP NCs increased from 2.7 to 12.8 nm with rising annealing temperature, accompanied by a broadening of the size distribution. This result underscores the critical role of thermal processing in tuning the structural properties of BP NCs.

### 4.3. Boron Phosphide Nanowires

One-dimensional (1D) nanowires, owing to their high aspect ratio and anisotropic charge transport channels, offer distinct advantages in reducing interfacial recombination losses and enhancing the separation efficiency of photogenerated carriers. BP, with its high carrier mobility [74], broad spectral response [29], and excellent thermal stability [42], can further leverage these benefits when integrated into a 1D architecture. This combination holds great promise for advanced optoelectronic applications, particularly in homojunction devices, photodetectors, and flexible sensing systems operating under extreme conditions.

**Figure 6:**
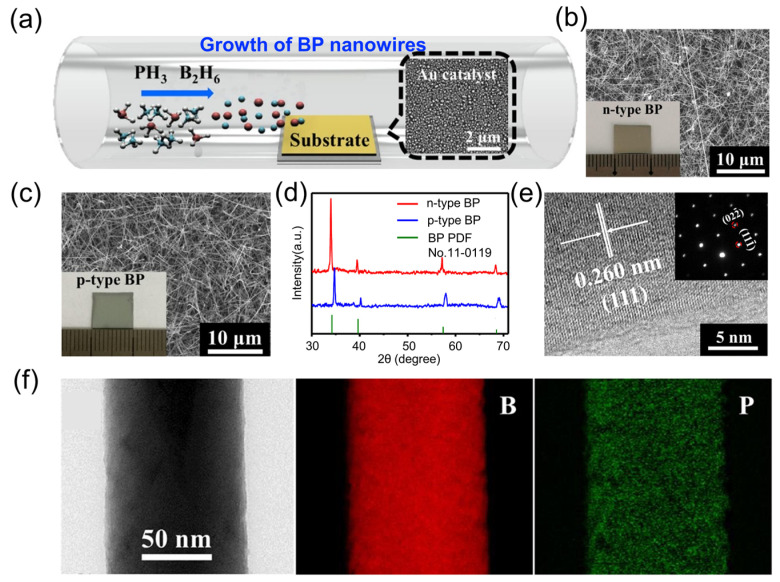
Synthesis of boron phosphide nanowires [44]. (**a**) Schematic diagram of CVD process showing controllable B/P precursor ratio through gas regulation, with inset illustrating Au catalyst distribution. (**b**) SEM image of *n*-type nanowires. (**c**) SEM image of *p*-type nanowires. (**d**) XRD patterns of *n*-type and *p*-type nanowires. (**e**) HRTEM image with inset showing diffraction pattern indicating nanowire growth along [111] direction. (**f**) EDX spectrum of a single nanowire. Reprinted with permission from [44], Copyright 2018, American Chemical Society.

In 2018, Zhai et al. [44] successfully synthesized 1D BP nanowires using a CVD method. By precisely controlling the B/P precursor ratio in the vapor phase, they achieved tunable carrier types in BP nanowires (Figure 6a). SEM images (Figure 6b,c) revealed that both *n*-type and *p*-type BP nanowires exhibited uniform diameters and extended lengths in the micrometer range. XRD analysis (Figure 6d) showed a noticeable blue shift in the diffraction peaks of *p*-type BP nanowires compared with their *n*-type counterparts, which was attributed to lattice strain induced by boron substitution at phosphorus sites. HRTEM images (Figure 6e) confirmed the lattice spacing of 2.60 Å, corresponding to the (111) crystallographic plane, while energy-dispersive X-ray (EDX) mapping (Figure 6f) demonstrated a homogeneous elemental distribution within individual nanowires.

**Figure 7:**
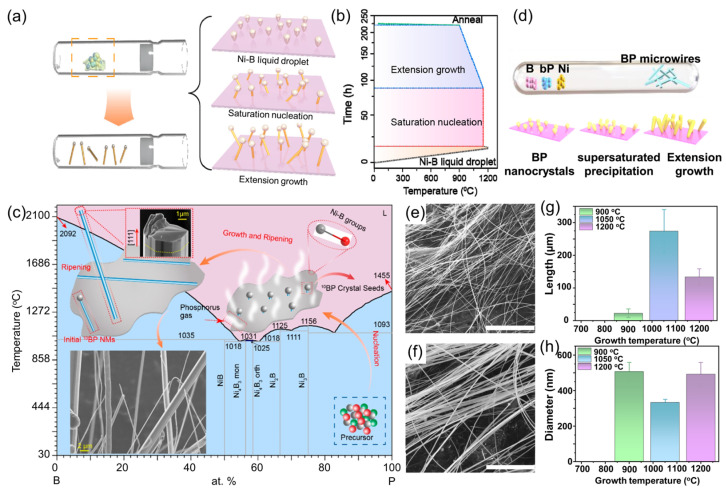
Synthesis of boron phosphide nanowires [50,52,87]. (**a**) Schematic of ^10^BP growth process. (**b**) Temperature profile used for the controlled growth of ^10^BP nanowires. Reprinted with permission from [50], Copyright 2022, American Chemical Society. (**c**) B-Ni phase diagram accompanied by a schematic of the growth process for ^nat^BP microfibers. Reprinted with permission from [52], Copyright 2023, American Chemical Society. (**d**) Schematic representation of BP microwire growth. (**e**) Low-magnification and (**f**) high-magnification SEM images of microwires grown at 1050 °C. Statistical analysis of the (**g**) length and (**h**) diameter distributions of BP microwires grown at different temperatures. Reprinted with permission from [87], Copyright 2025, Elsevier Inc.

Building on these advancements, Zheng et al. [50,52] expanded the synthetic strategies for BP 1D micro/nanostructures using a vapor–liquid–solid (VLS) method (Figure 7a,b). By introducing nickel catalysts and optimizing temperature control, this study successfully addressed the challenge posed by the high melting point of boron and the low sublimation temperature of phosphorus. The resulting ^10^BP microwires exhibited an exceptionally high aspect ratio of 10^4^, demonstrated rapid carrier migration along the growth direction, and displayed an average hardness of 41 GPa in nanoindentation tests. High-pressure in situ XRD confirmed their stability and reversible structural recovery under pressures up to 36 GPa.

The growth mechanism of BP 1D micro/nanostructures was further elucidated through the B-Ni phase diagram (Figure 7c). Pure-phase natural boron phosphide (^nat^BP) single-crystal microwires were synthesized using the same VLS method. At temperatures above 1000 °C, B-Ni alloy formation facilitated phosphorus diffusion, leading to BP crystal nucleation, followed by directional growth along [111] during slow cooling. Notably, the synthesized ^nat^BP microwires exhibited extraordinary elastic deformation capabilities, with bending angles reaching up to 180°, laying a crucial foundation for their potential application in flexible electronic devices.

More recently, Su et al. [87] synthesized high-density BP micro/nanowires via a vapor–solid (VS) growth mechanism (Figure 7d). Systematic temperature-dependent studies identified 1050 °C as the optimal growth condition, yielding BP micro/nanowires with lengths exceeding 250 μm and diameters of 250–350 nm (Figure 7e–h). Notably, these BP micro/nanowires exhibited exceptional environmental stability, retaining their crystalline structure without noticeable degradation even after six months of exposure to ambient air.

These advancements not only deepen the understanding of BP nanowire growth mechanisms but also establish a robust platform for high-performance BP-based electronic and optoelectronic devices. Future efforts should focus on precise doping strategies and heterostructure engineering to further tailor their electronic properties, unlocking their full potential for next-generation semiconductor applications.

### 4.4. Boron Phosphide Films

Two-dimensional BP films have garnered significant interest because of their unique electrical and optical properties, positioning them as promising candidates for next-generation optoelectronic devices. Early studies primarily explored epitaxial growth on silicon and sapphire substrates [88,89,90,91,92]. However, the substantial lattice mismatch in these systems frequently introduces unintended impurities and structural defects that compromise device performance. To overcome these challenges, researchers have begun investigating alternative substrates, such as SiC, AlN, and ZrB_2_, which offer better lattice matching and facilitate the growth of higher-quality BP films.

Padavala et al. [43] successfully deposited BP films on 3C-SiC substrates via the CVD method, achieving superior crystallinity and a pronounced preferred orientation. Notably, this work revealed that lower deposition temperatures (1000–1100 °C) enhance crystal orientation and surface morphology, whereas higher temperatures tend to induce the formation of polycrystalline phases. These findings underscore the critical role of substrate selection and thermal control in optimizing film quality.

**Figure 8:**
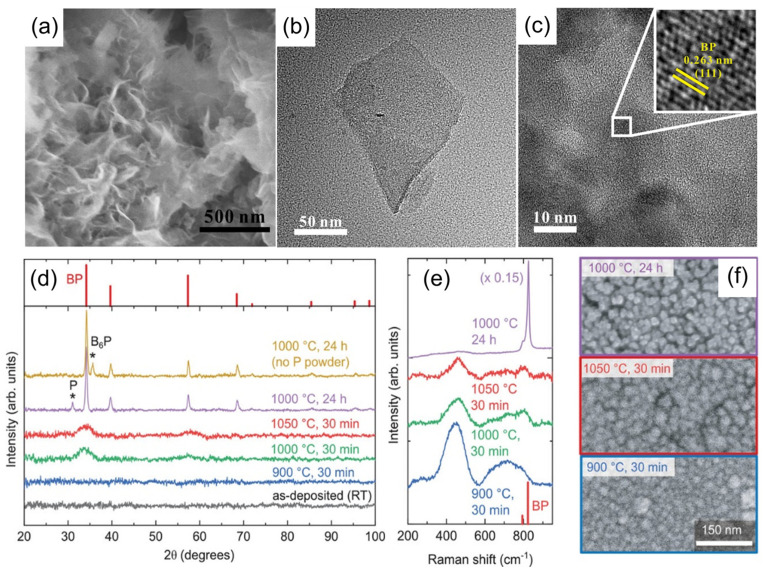
Synthesis of boron phosphide thin films [40,49]. (**a**) SEM image of BP nanosheets. (**b**) TEM image of BP nanosheets after ultrasonic treatment. (**c**) HRTEM image of BP nanosheets with an inset highlighting the lattice fringes to illustrate crystallinity. Reprinted with permission from [49], Copyright 2020, Elsevier Ltd. (**d**) XRD patterns of various C-doped BP films annealed in sealed ampoules. (**e**) Raman spectra of four BP films as shown in (**d**). (**f**) SEM images of three BP films as depicted in (**d**). Reprinted with permission from [40], Copyright 2022, Wiley-VCH.

Subsequently, Li et al. [49] reported the fabrication of BP nanosheets with thicknesses of approximately 4 nm via a SiO_2_-assisted solid-state reaction. In this method, a homogeneous mixture of boron, phosphorus, and SiO_2_ nanoparticles was ground, sealed in a quartz ampoule, and annealed. SEM and TEM analyses (Figure 8a–c) confirmed that the produced BP nanosheets were ultrathin and exhibited variable lateral dimensions, while HRTEM images revealed a lattice spacing of 0.263 nm corresponding to the (111) planes of cubic BP, with SAED further verifying the crystal structure.

BP film synthesis remains challenging because of the high inertness of boron and the volatility of phosphorus, necessitating growth temperatures above 900 °C, which pose a risk of material decomposition. Conventional CVD techniques address this by employing high phosphorus partial pressures to suppress evaporation. In contrast, sputtering deposition, which enables high dopant solubility at moderate pressures, has emerged as a promising alternative. In 2022, Crovetto et al. [40] pioneered the preparation of amorphous BP films via the reactive sputtering method. In the approach, PH_3_ was introduced as a reactive gas during sputtering from a boron target, resulting in amorphous BP films that were subsequently crystallized through annealing in a phosphorus-rich atmosphere at 1000 °C. The enhanced crystallinity of these films was confirmed by XRD, Raman spectroscopy, and SEM (Figure 8d–f).

Collectively, these studies highlight the evolving strategies to optimize BP film growth—whether through substrate engineering, alternative synthesis routes, or innovative deposition techniques—and pave the way for the integration of high-quality 2D BP films into advanced electronic and photonic devices.

To provide a comprehensive comparison, Table 2 systematically compares the advantages, limitations, and yield or size data for key boron phosphide (BP) synthesis methods, including chemical vapor deposition (CVD), high-pressure co-crystal melting, molten salt, and vapor–liquid–solid (VLS) approaches. Notably, gaps in yield data across several methods remain, underscoring the need for further optimization and standardized reporting in future research.

## 5. Applications of Boron Phosphide

With the successful development of high-quality BP micro- and nanostructures, a new era in advanced functional materials research has emerged. The rigorous synthesis techniques described above have enabled precise control over BP’s crystalline phases, morphology, and electronic properties, establishing an ideal platform for a wide range of emerging applications. Owing to its wide bandgap, as well as its exceptional thermal and chemical stability, BP is a promising candidate for next-generation devices in detection, sensing, energy conversion, and energy storage.

### 5.1. Boron Phosphide for Photodetectors and Sensors

**Figure 9:**
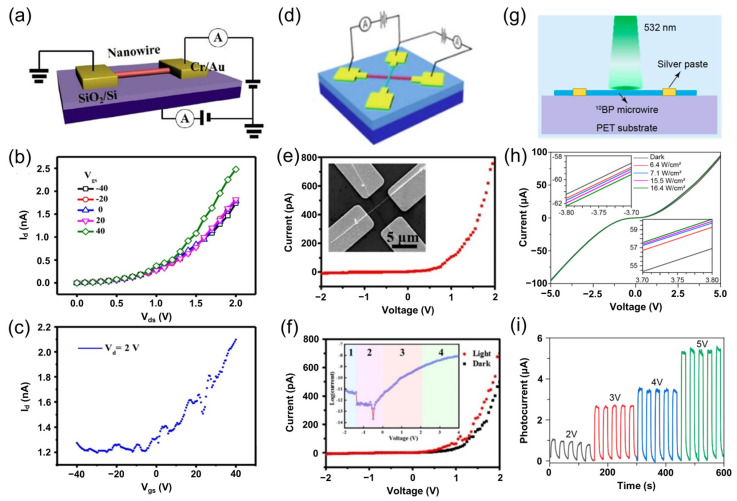
BP-based photodetectors [44,50]. (**a**) Schematic of the device structure for field-effect measurements. (**b**) Output characteristics and (**c**) transfer characteristics of a *n*-type BP nanowire transistor. (**d**) Schematic of a crossed homojunction formed by *n*-type and *p*-type BP nanowires. (**e**) Rectification behavior of the homojunction, with an inset showing an SEM image of the cross-junction device. (**f**) *I*–*V* characteristics of the homojunction under dark and illuminated conditions, accompanied by a semilog plot to highlight different carrier transport regimes. Reprinted with permission from [44], Copyright 2018, American Chemical Society. (**g**) Schematic of a photoconductive detector fabricated on a flexible substrate. (**h**) *I*–*V* curves of a single ^10^BP nanowire photodetector under varying light intensities (insets: magnified *I*–*V* plots under forward and reverse biases between 3.7–3.8 V). (**i**) Photocurrent time responses under 532 nm light (15.5 W/cm^2^) at various bias voltages. Reprinted with permission from [50], Copyright 2022, American Chemical Society.

As detailed in the preceding BP nanowire synthesis section, Ding et al. [44] demonstrated that the carrier type in BP nanowires can be effectively tuned by adjusting the boron-to-phosphorus precursor ratio during CVD. This tunability enables the construction of well-defined homojunction and high-performance optoelectronic devices. Building on this approach, BP nanowires were integrated into FETs and homojunction photodetectors, as illustrated in Figure 9a.

Electrical characterization of an *n*-type BP nanowire transistor (Figure 9b,c) revealed typical electron-dominated conduction, with output (*I*_ds_–*V*_ds_) and transfer (*I*_ds_–*V*_gs_) curves indicating a carrier mobility of approximately 0.093 cm^2^·V^−1^·s^−1^. In addition, a crossed homojunction was also constructed by integrating *n*-type and *p*-type BP nanowires using transfer techniques (Figure 9d). Electrical measurements of the homojunction exhibited pronounced rectification behavior with a rectification ratio of about 100 (Figure 9e), and the SEM inset confirmed the well-defined microstructure of the crossed device.

Under illumination, the current–voltage (*I*–*V*) characteristics of the *p*-*n* junction device showed significant modulation compared with dark conditions (Figure 9f), confirming its optoelectronic sensitivity. Recently, Zheng et al. [50] investigated the photoresponse of 1D ^10^BP nanowires (Figure 9g). The device exhibited a relatively high dark current (~90 μA at a 5 V bias) that increased with light intensity (Figure 9h), indicating strong optical excitation sensitivity. Although reproducible photoresponses were observed under various light intensities and bias conditions (Figure 9i), the large dark current partially obscured the photocurrent enhancement.

**Figure 10:**
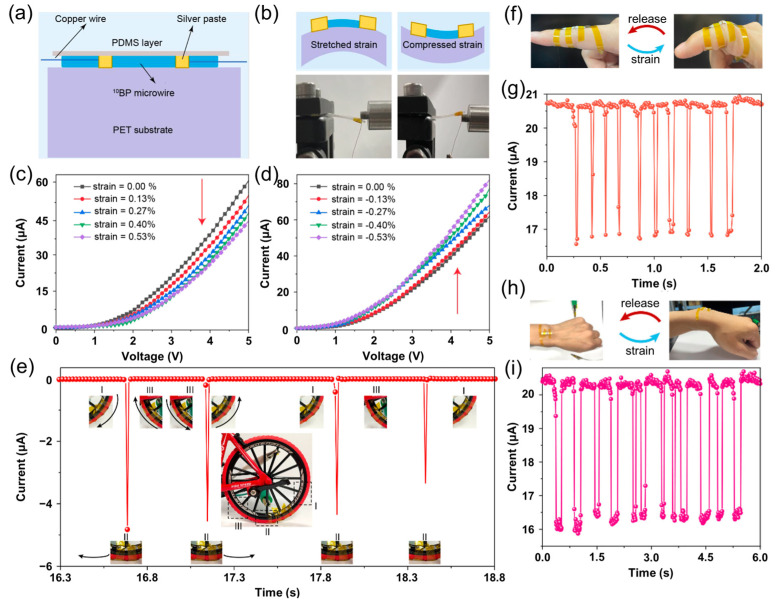
Applications of BP in flexible sensing [50,52]. (**a**) Schematic of a strain sensor based on a single ^10^BP micro-/nanowire. (**b**) Schematic and optical images of the ^10^BP sensor in stretched and compressed states. (**c**) *I*–*V* characteristics under various tensile strains. (**d**) *I*–*V* characteristics under various compressive strains. Reprinted with permission from [50], Copyright 2022, American Chemical Society. (**e**) Current response during tire rotation. (**f**) Photograph showing finger flexion monitoring. (**g**) Current signals corresponding to finger bending and extension at 3 V bias. (**h**) Photograph of wrist monitoring. (**i**) Current signals corresponding to wrist bending and extension at 3 V bias. Reprinted with permission from [52], Copyright 2023, American Chemical Society.

Beyond optoelectronic applications, Zheng et al. [50] explored piezoelectric detection using the ^10^BP nanowires. A device was fabricated by fixing electrodes at both ends of a ^10^BP microwire on a Polyethylene Terephthalate (PET) substrate using a silver paste, followed by encapsulation in Polydimethylsiloxane (PDMS) (Figure 10a,b). Under applied tensile strain, the *I–V* curve shifted downward, whereas compressive strain induced an upward shift, with full recovery upon strain release. The current decreased with increasing tensile strain (Figure 10c) and increased under compression (Figure 10d), demonstrating excellent reversibility and repeatability.

Based on this, Zheng et al. [52] demonstrated the application of BP microwire-based flexible strain sensors in bicycle speed detection and motion monitoring. Using a polyimide (PI) substrate, the sensor conforms closely to the curvature of a bicycle tire (Figure 10e). During tire rotation, the sensor produces electrical signals corresponding to the strain induced by deformation. In wearable applications, sensors fixed on a finger (Figure 10f) and wrist (Figure 10h) generated reproducible and stable electrical signals (Figure 10g,i), with current decreasing upon bending and recovering when extended. These promising results underscore the potential of BP-based sensors for applications in wearable electronics, human–machine interfaces, and soft robotics.

### 5.2. Boron Phosphide for Thermal Management

As electronic and photonic devices continue to miniaturize and integrate at high densities, there is an increasing demand for polymer-based composites with enhanced thermal conductivity. Although these materials exhibit exceptional heat transfer properties, they come with notable drawbacks [67]. For example, diamond—while already the most developed material for passive cooling in high-power electronics—suffers from high cost, slow synthesis rates, variable quality, and challenges in integration with semiconductor components. In addition, graphene and carbon nanotubes also offer extraordinary thermal conductivity at the nanoscale; however, their performance tends to degrade when assembled into practical devices because of ambient interactions and disorder scattering, and their intrinsic anisotropy further complicates device applications. Graphite and carbon black show low cost and processability, but the high electrical conductivity of these carbon-based materials still leads to potential short-circuit risks when used in thermal management roles.

In contrast, BP exhibits a moderate thermal conductivity, typically reported at approximately 500 W/m·K, but its pronounced isotropic properties enable uniform heat dissipation in all directions. Additionally, as a semiconductor, BP inherently avoids the electrical conduction issues associated with carbon materials, thereby offering a promising balance between effective thermal management and safe integration in devices [47,76].

**Figure 11:**
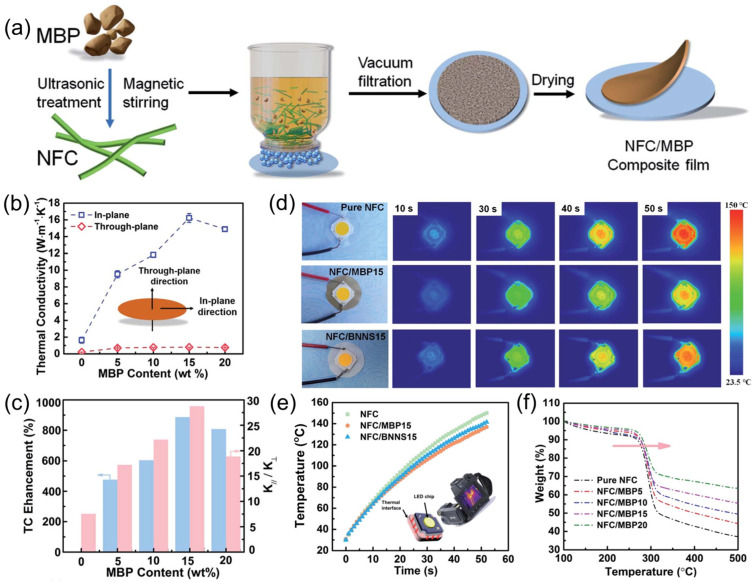
Applications of BP in thermal management [47]. (**a**) Diagram depicting the step-by-step fabrication process of NFC/MBP composite films. (**b**) Measured thermal conductivity values for NFC/MBP composite films. (**c**) Trends in thermal conductivity improvement and anisotropy ratios for NFC/MBP composite films as MBP content changes. (**d**) Infrared (IR) thermal images of pure NFC, NFC/MBP15, and NFC/BNNS15 films at various heating intervals. (**e**) Surface temperature changes over time for LED chips attached to pure NFC, NFC/MBP15, and NFC/BNNS15 films. (**f**) Thermogravimetric analysis results for NFC and NFC/MBP composite films. Reprinted with permission from [47]. Copyright 2021, Royal Society of Chemistry.

Hu et al. [47] synthesized BP powder via a molten salt method and then subjected it to high-energy ball milling to produce milled boron phosphide (MBP) particles rich in surface functional groups. As illustrated in Figure 11a, MBP particles were mixed with nanofibrillated cellulose (NFC) and subsequently processed into composite films by vacuum filtration. At an MBP content of 15 wt% (NFC/MBP15), the in-plane thermal conductivity of the composite film increased dramatically from 1.63 W·m^−1^·K^−1^ (pure NFC film) to 16.09 W·m^−1^·K^−1^ (Figure 11b), an 887% enhancement over pure NFC (Figure 11c). This improvement, however, is highly anisotropic—while the in-plane conductivity is significantly elevated, the through-plane conductivity exhibits only a moderate increase. Such anisotropy arises from the layered structure of NFC, where MBP particles form short-range ordered heat conduction pathways via hydrogen bonding.

Beyond enhancing thermal conductivity, the NFC/MBP composite film also demonstrated superior heat dissipation performance when employed as a flexible substrate for LED chips (Figure 11d–f). Under identical conditions, after 50 s of operation, the center temperature of an LED chip mounted on the NFC/MBP15 composite was 134.5 °C, which is lower than the 145.2 °C observed for pure NFC and 138.5 °C for NFC/BNNS15 (nanofibrillated cellulose/boron nitride nanosheets). The composite films also exhibited excellent thermal stability, flexibility, mechanical strength, and moderate electrical insulation. Thermogravimetric analysis (Figure 11f) showed that while pure NFC suffers significant weight loss between 255 and 312 °C because of the elimination of side groups, the T_10%_ (the temperature at which the material loses 10% of its initial mass during heating) value increases from 268 °C to 281 °C upon MBP incorporation.

In parallel, He et al. [93] developed a novel 3D heat transfer network (3D-BP@Ni) by in situ growth of BP grains on nickel foam, which significantly improved the thermal conductivity of epoxy resin composites. The integration of 3D-BP@Ni led to a remarkable 908.53% increase in thermal conductivity while effectively reducing the coefficient of thermal expansion to 26.95 × 10^−6^/°C.

The result demonstrates that BP exhibits tremendous potential in thermal management, particularly when employed as a filler in polymer-based composites. By optimizing fabrication processes and tailoring material combinations, the intrinsic properties of BP can be further enhanced to meet the demanding requirements of high-performance thermal management materials in advanced electronics. Moreover, the synergistic integration of BP with complementary fillers and the application of effective interface engineering provides a promising pathway toward next-generation thermal management solutions. These findings not only underscore the importance of precise synthetic control but also offer a robust foundation for future developments in high-efficiency thermal management technologies.

### 5.3. Boron Phosphide for Energy Conversion

In addition to its applications in sensor fabrication and thermal management, BP has garnered significant attention in catalysis owing to its high specific surface area, abundant surface-active sites, and exceptional chemical stability [29,48,53]. These properties have been leveraged in various catalytic processes, with BP nanoparticles emerging as promising candidates for photocatalytic hydrogen production and CO_2_ reduction.

**Figure 12:**
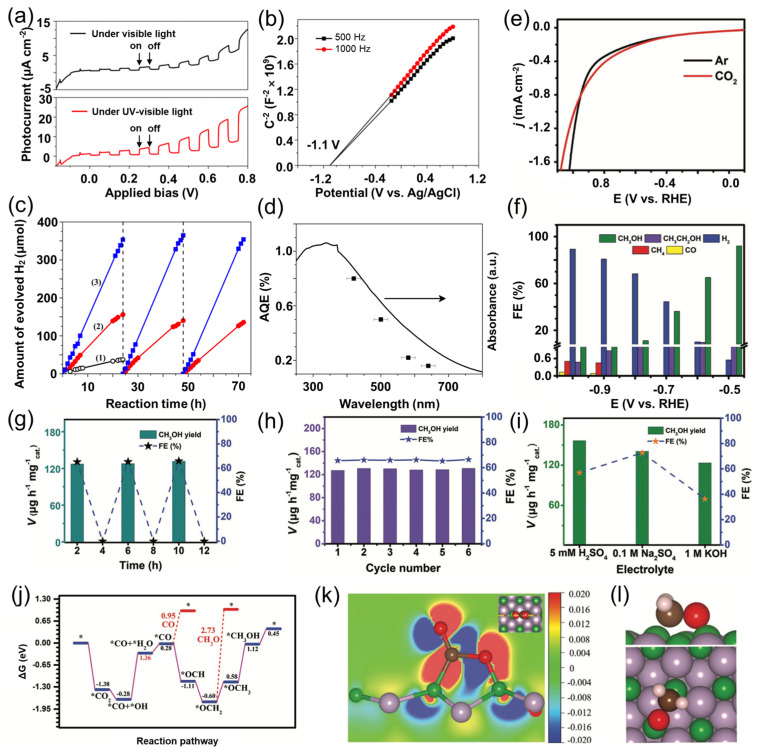
Applications of BP in energy conversion [29,53]. (**a**) Photocurrent response of BP as a function of applied bias under UV-vis and visible light illumination. (**b**) Mott–Schottky analysis of BP. (**c**) Hydrogen production kinetics from water containing 10 vol% formaldehyde as a sacrificial agent under visible light irradiation, comparing unmodified BP, 1 wt% Pt-loaded BP, and 1 wt% Pt-loaded ball-milled BP photocatalysts. (**d**) UV-vis diffuse reflectance spectra and AQE of 1 wt% Pt-loaded BP, using formaldehyde as the electron donor. Reprinted with permission from [29], Copyright 2016, Elsevier Ltd. (**e**) LSV curves of BP/CP in Ar- and CO_2_-saturated 0.1 M KHCO_3_ electrolyte. (**f**) FEs of BP/CP at various applied potentials in CO_2_-saturated 0.1 M KHCO_3_ electrolyte. (**g**) Methanol production rates and corresponding FEs for BP/CP during 2-h cycles in CO_2_- and Ar-saturated electrolytes. (**h**) Methanol yields and FEs at −0.6 V for recycling tests in 0.1 M KHCO_3_. (**i**) Methanol production performance at −0.6 V in different electrolyte environments. (**j**) Free energy profile for CO_2_RR on the BP (111) surface. The asterisk (*) denotes a species adsorbed on the catalyst surface. (**k**) Electron density distribution following CO_2_ adsorption on the BP (111) surface, with red and blue regions indicating electron accumulation and depletion, respectively (isosurface level is 0.02 e Bohr^−3^). (**l**) Atomic configuration of the *OCH_2_ intermediate, with color codes: B (green), P (gray), C (brown), O (red), H (white). Reprinted with permission from [53], Copyright 2019, Wiley-VCH.

Shi et al. [29] demonstrated the catalytic potential of BP nanoparticles in hydrogen evolution reactions (HER), revealing their unique advantages in photocatalysis. BP exhibits an indirect band gap of 2.0 eV, which enables it to effectively absorb visible light in the 300–800 nm range, thereby driving photocatalytic reactions. Initially, photocurrent response tests and Mott–Schottky (M-S) measurements were performed to elucidate BP’s electrical characteristics. As shown in Figure 12a,b, the photocurrent increases with an increasing positive bias, and the M-S plots display a positive slope, indicating that BP is a *n*-type semiconductor. This *n*-type behavior underpins BP’s outstanding photocatalytic activity, enabling stable hydrogen generation in aqueous solutions containing sacrificial electron donors such as formaldehyde, even without noble metal co-catalysts. As shown in Figure 12c (curve 1), the initial hydrogen evolution rate of the material is recorded at 2.0 μmol·h^−1^. Upon the incorporation of 1 wt% Pt, the photocatalytic hydrogen production rate significantly improves, reaching 6.9 μmol·h^−1^ (curve 2). This increase highlights the beneficial role of Pt as a co-catalyst in enhancing photocatalytic performance. While the intrinsic activity of BP-based photocatalysts is typically lower than that of their metal-containing counterparts, the reduction of BP crystal size through mechanical ball milling is found to boost photocatalytic hydrogen evolution further. This size reduction improves the separation efficiency of photogenerated electron-hole pairs, thereby promoting the hydrogen evolution reaction under visible light. Over a 72-h period, a total hydrogen evolution of 1072 μmol is achieved (curve 3), underscoring the remarkable photocatalytic activity of the ball-milled BP-based catalyst. Furthermore, wavelength-dependent apparent quantum efficiency (AQE) measurements reveal that the AQE decreases with increasing incident light wavelength, which is consistent with the visible light absorption spectrum and confirms that the hydrogen generation is indeed photo-driven (Figure 12d).

Mou et al. [53] investigated the potential of BP nanoparticles for CO_2_ reduction, demonstrating their ability to convert CO_2_ to methanol efficiently. As shown in Figure 12e, in a 0.1 M KHCO_3_ electrolyte, when a potential of −0.5 to −1 V is applied, a higher current density is observed in the CO_2_-saturated electrolyte, indicating that a CO_2_ reduction reaction occurs. At −0.5 V, BP nanoparticles achieve a methanol Faradaic efficiency as high as 92.0% (Figure 12f). In Figure 12g, CH_3_OH is detected only in CO_2_-saturated electrolytes, confirming that the methanol originates from the electrocatalytic CO_2_RR on the BP/CP interface. Stability tests further show that at −0.6 V, the methanol production rate remains stable at 127.5 μg·h^−1^·mg^−1^_cat_ (Figure 12h), and high CO_2_-to-CH_3_OH conversion activity is maintained over a broad pH range (Figure 12i). Moreover, DFT calculation results reveal the reaction mechanism for CO_2_ reduction on the BP (111) surface. Their results indicate that the cooperative effect of boron and phosphorus enhances CO_2_ adsorption and activation, with the rate-determining step being the transformation from *CO + *OH to *CO + *H_2_O accompanied by a free energy change of 1.36 eV (Figure 12j). The electron density difference map in Figure 12k clearly shows that upon CO_2_ adsorption on BP (111), electrons are transferred from phosphorus to boron, thus activating the CO_2_ molecule. Furthermore, Figure 12l illustrates that the *OCH_2_ intermediate is adsorbed on BP (111) via side coordination, and owing to its high desorption energy, it is more inclined to undergo further hydrogenation to form CH_3_OH rather than CH_2_O. This explains the high selectivity for methanol production in the CO_2_RR catalyzed by BP nanoparticles. The excellent structural and electrochemical stability demonstrated by BP nanoparticles during prolonged electrocatalysis further underscores their practical potential in the field of electrocatalysis.

### 5.4. Boron Phosphide for Energy Storage

Due to its unique electronic structure and high conductivity, BP has also attracted significant attention in the field of energy storage [51,94,95,96,97,98,99]. Recent advances illustrate the versatility of BP-based nanostructures in both experimental devices and theoretical investigations.

**Figure 13:**
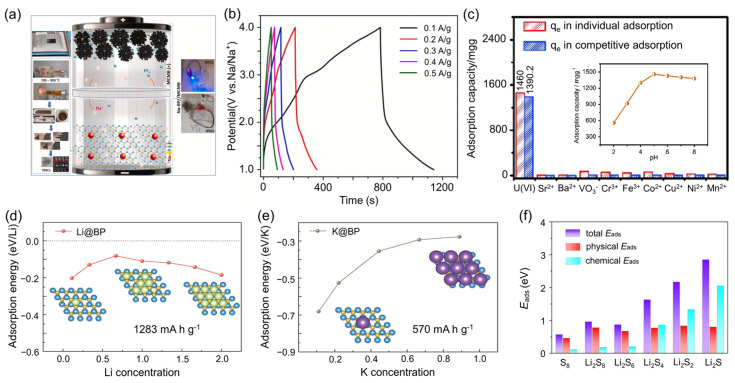
Applications of BP in energy storage [51,95,96,97]. (**a**) Schematic and technical photographs illustrating the synthesis of Na-BP and the assembly of the Na-BP//MCMB’ device for NISCs. (**b**) GCD profiles of the Na-BP-900//MCMB device at current densities ranging from 0.1 to 0.5 A/g. Reprinted with permission from [51], Copyright 2023, Elsevier Ltd. (**c**) Equilibrated adsorption capacity (qe) of individual and competitive metal ions in aqueous solution: UO_2_ (100 mg/L) and other metal ions (1 × 10^4^ mg/L). The inset displays the electrosorption capacities of U(VI) ions at varying pH levels. Reprinted with permission from [95], Copyright 2020, Elsevier B.V. Adsorption energy changes as the function of (**d**) Variation in adsorption energy as a function of Li concentration in Li_x_BP, with embedded figures showing the top-view optimized structures of key intermediates. (**e**) Variation in adsorption energy as a function of K concentration in K_x_BP, with embedded figures depicting the top-view optimized structures of key intermediates Reprinted with permission from [96], Copyright 2017, Royal Society of Chemistry. (**f**) Total adsorption energy, including both physical and chemical contributions, for the S_8_ and Li_2_S_n_ (n = 8, 6, 4, 2, and 1) species on a BP monolayer. Reprinted with permission from [97], Copyright 2019, Elsevier B.V.

Thirumal et al. [51] synthesized sodium–boron phosphide (Na-BP) nanosheets via a facile thermal annealing route, demonstrating their potential as high-performance anodes in sodium-ion hybrid supercapacitors (NISCs). Paired with mesoporous carbon microspheres (MCMB) as the positive electrode, Na-BP nanosheets enable a broad operating voltage range of 1–4 V. As shown in Figure 13a, the Na-BP nanosheets exhibit excellent electrochemical stability. Notably, Figure 13b displays the galvanostatic charge–discharge (GCD) curves of the Na-BP-700//MCMB sodium-ion supercapacitor (NISC) device under a series of current densities: 0.1, 0.2, 0.3, 0.4, and 0.5 A/g. All curves exhibit nearly linear and symmetric charge–discharge profiles within the working voltage window of 1–4 V, indicative of typical electrochemical double-layer capacitive (EDLC) behavior with minimal faradaic contributions. The elimination of a pre-sodiation step further streamlines the fabrication process, underscoring the potential for scalable and cost-effective device manufacturing.

In addition, BP has also been explored for capacitive deionization (CDI). Chen et al. [95] employed amorphous BP nanosheets for the selective extraction of uranium (U(VI)) ions, demonstrating remarkable selectivity even in the presence of a 100-fold excess of competing ions such as Sr^2+^, Ba^2+^, and VO_3_^−^ (Figure 13c). The electrosorption capacity reaches its peak near the pH value of 5, underscoring the tunable surface chemistry of BP for targeted ion recovery applications.

Theoretical investigations have further expanded the potential scope of BP in energy storage. Hexagonal BP, predicted to possess robust structural stability and distinctive electronic properties, holds promise as an advanced anode material. First-principles calculations reveal that BP monolayers could provide exceptional performance in alkali metal (Li/Na/K) batteries [96]. At low Li concentrations, repulsive interactions between adjacent Li atoms reduce the adsorption energy, whereas at higher concentrations, structural rearrangements in the BP lattice enhance the adsorption energy (Figure 13d). In contrast, potassium adsorption shows a continuous decrease in adsorption energy until full bilayer coverage is achieved (Figure 13e). The corresponding theoretical specific capacities, which are 1283 mA·h·g^−1^ for Li and 570 mA·h·g^−1^ for K, significantly surpass the 372 mA·h·g^−1^ offered by conventional graphite anodes, thereby underscoring the high energy density potential of BP-based electrodes. Moreover, the adsorption process induces a semiconductor-to-metal transition in BP, markedly enhancing its electrical conductivity and ensuring efficient charge transport during battery cycling.

Beyond its application as a negative electrode, BP monolayers have also been explored as anchoring substrates in lithium–sulfur (Li-S) batteries. Yu et al. [97] demonstrated that higher-order polysulfides (e.g., Li_2_S_8_ and Li_2_S_6_) interact with BP predominantly by van der Waals forces, while lower-order polysulfides (e.g., Li_2_S_2_ and Li_2_S) form stronger chemical bonds with the BP surface (Figure 13f). This interaction leads to a reduction in the band gap and an enhancement in conductivity, which promotes rapid electron transfer. As a result, the shuttle effect is effectively suppressed, leading to improved overall battery performance.

Collectively, these studies highlight the multifaceted potential of boron phosphide. Its proven applications in sodium-ion hybrid supercapacitors and capacitive deionization for uranium separation, coupled with promising theoretical predictions for Li- and K-ion batteries and Li-S systems, establish BP as a compelling material for next-generation energy storage and conversion technologies. Future research is anticipated to optimize the synthesis and integration of BP nanostructures, paving the way for advanced applications in hybrid electric vehicles, smart grid storage, and even seawater uranium extraction, thereby addressing both energy and environmental challenges with high-performance materials.

## 6. Summary and Outlook

### 6.1. Summary

This review provides a comprehensive evaluation of BP, elucidating its unique structure, exceptional performance in various domains, and recent advances in synthesis techniques and functional applications. BP exhibits high hardness, ultra-high thermal conductivity, outstanding chemical stability, and mechanical flexibility. Its wide bandgap makes it an ideal material for high-performance electronic devices, optoelectronic systems, and energy conversion platforms. Notably, by employing dimensional engineering from bulk crystals to nanoparticles, nanowires, and 2D films, alongside doping strategies, the electrical properties of BP can be finely tuned to meet the diverse demands of extreme thermal management and flexible device applications. The key conclusions are as follows:

1. Progress in synthesis and methodological innovations

Considerable progress has been made in the synthesis of BP, ranging from bulk single crystals to precisely engineered low-dimensional nanostructures. Innovative techniques, such as high-pressure co-crystal melting and nickel-catalyzed molten salt methods, have significantly enhanced yield and uniformity, as well as enabled directional growth and performance optimization. These advancements mark a major milestone in BP synthesis, paving the way for deeper investigations into its intrinsic properties and broad application potential.

2. Applications of boron phosphide in various fields

BP’s structural versatility enables outstanding performance across multiple fields. BP nanowires, with their high carrier mobility and mechanical elasticity, have achieved breakthroughs in photodetectors and wearable sensors. In the realm of thermal management, BP’s inherent high thermal conductivity and stability position it as an ideal candidate for advanced heat dissipation systems. In energy conversion and storage, BP nanoparticles, with their high surface area, abundant active sites, and tunable electronic states, significantly enhance catalytic efficiency for processes such as HER, oxygen reduction reaction (ORR), and photoinduced chemical conversions. Additionally, amorphous BP nanosheets exhibit remarkable selectivity for uranium (VI) ions, highlighting their potential for nuclear resource recovery. BP-based composite electrodes also demonstrate enhanced ion-insertion kinetics, making them promising candidates for supercapacitors and batteries.

### 6.2. Future Challenges and Outlook

1. Synthesis and scalability challenges

The controlled synthesis of BP, whether in its cubic (*c*-BP) or hexagonal (*h*-BP) phase, remains a key bottleneck toward widespread application. This challenge stems from the volatility of phosphorus and the high melting point of boron, which require stringent reaction conditions. Current high-yield methods—such as the vapor–liquid–solid method and chemical vapor deposition—often operate under high temperatures (>1000 °C) or rely on tightly controlled precursor ratios, limiting their scalability and energy efficiency. For instance, achieving stoichiometric purity in CVD-grown nanostructures is complicated by non-uniform P:B ratios and oxygen contamination, both of which degrade device performance and contribute to variability in reported electronic properties.

To address these limitations, molten-salt-assisted CVD has emerged as a promising approach analogous to methods developed for *h*-BN synthesis. This strategy enables the stabilization of reactive intermediates and lowers energy barriers for crystal growth. Additionally, substrate engineering can reduce interfacial strain and promote epitaxial alignment, improving film quality over large areas. Real-time process monitoring using machine learning-driven gas-phase diagnostics offers another frontier, enabling better kinetic control and reduced defect formation. Such advancements, coupled with standardized synthesis protocols, are essential to transition BP from lab-scale demonstrations to industrial-scale technologies.

2. Theoretical and computational frontiers

First-principles calculations have laid a solid foundation for understanding the intrinsic electronic, optical, and thermal properties of BP. However, most existing models consider idealized, defect-free structures and neglect the impact of point defects, grain boundaries, and interfacial interactions—factors that critically affect device-level behavior. For instance, phosphorus vacancies in BP are predicted to act as electron traps, yet their quantitative effect on carrier lifetimes, especially in optoelectronic devices such as photodetectors, remains unexplored.

To bridge this gap, multiscale simulations that couple density functional theory (DFT) with molecular dynamics or mesoscale modeling are needed to capture dynamic defect formation, phonon scattering, and chemical degradation under operational conditions such as thermal cycling and mechanical bending. Furthermore, although BP exhibits relatively isotropic thermal conductivity, a deeper theoretical framework linking phonon transport to macroscopic heat dissipation is lacking. This knowledge would guide the rational design of BP-based composites—such as BP-SiC hybrids—with optimized thermal properties for use in high-power or flexible electronics.

3. Material performance and application gaps

While BP exhibits a wide range of functional capabilities—ranging from electrocatalysis and sensing to energy storage and optoelectronics—there are still substantial gaps in both mechanistic understanding and application reliability. For example, BP’s catalytic activity for hydrogen evolution and CO_2_ reduction is thought to arise from charge redistribution at polar B–P bonds, diverging from traditional transition-metal-based mechanisms. However, operando studies (e.g., in situ X-ray absorption or infrared spectroscopy) under real working conditions are needed to confirm these hypotheses and optimize active site design.

In optoelectronics, CVD-grown BP nanowires have demonstrated high carrier mobility, but their performance often varies due to inconsistent stoichiometry and device fabrication techniques. Additionally, while BP’s chemical stability in acidic and basic environments has been verified, its long-term reliability under realistic operational stressors—such as UV exposure, cyclic mechanical loading, and repeated charge/discharge cycles—remains insufficiently characterized.

4. Interdisciplinary opportunities inspired by 2D Materials

The recent progress of 2D materials such as graphene and *h*-BN provides a valuable blueprint for the development of BP-based systems. For instance, van der Waals integration of BP with TMDCs can create heterostructures with tailored band alignments, enabling multifunctional optoelectronic devices. Additionally, BP’s intrinsic resistive switching properties hold promise for neuromorphic computing but remain largely unexplored. Techniques such as spatiotemporal photoconductivity mapping—pioneered in MoS_2_ synaptic devices—could reveal BP’s potential for bioinspired electronics.

In the field of thermal management, BP-epoxy composites mirror early challenges seen in graphene-polymer systems. Enhancing thermal conductivity through optimized filler morphology, aspect ratio, and interfacial functionalization (e.g., phosphonate coupling) may improve phonon percolation pathways and device-level performance. Furthermore, the development of low-cost, inkjet-compatible BP inks is essential for printed electronics and flexible sensors yet remains technically challenging because of BP’s reactivity and processing sensitivity.

Sustainability is a cross-cutting issue that must be prioritized as BP moves toward commercialization. The environmental burden of high-temperature synthesis and the potential ecotoxicity of BP nanoparticles necessitate comprehensive life-cycle assessments. In parallel, phosphorus recycling strategies—from waste streams or end-of-life devices—could be integrated to support circular manufacturing paradigms.

## Figures and Tables

**Table 1:** Comparison of thermal, mechanical, and electronic properties of boron phosphide and representative semiconductors.

Material	Thermal Conductivity (W/m·K)	Hardness (GPa)	Electron Mobility (cm^2^/V·s)	Hole Mobility (cm^2^/V·s)	Bandgap (eV)	Notes
**BP**	**~500**	**38**	**140**	**500**	**~2.0**	**Indirect gap**
Si [81]	149	13	1400	450	~1.12	Indirect gap
Bas [82]	1300	22	1400–1740	1600	~1.82	Indirect gap
GaAs [83]	45	5.5	8500	400	~1.42	Direct gap
GaP [84]	75.2	8.8	250	150	~2.24	Indirect gap
InP [85]	68	5.0	<5400	<200	~1.35	Direct gap
GaN [86]	130	10.2	<1000	<350	~3.4	Direct gap

**Table 2:** Comparison of boron phosphide synthesis methods.

Method	Morphology	Conditions	Advantages	Limitations	Yield/Size Control	Ref.
**High-pressure co-crystal melting**	Bulk	5.0 GPa, 3000 °C	Produces millimeter-scale single crystals	Extreme conditions limit scalability	≤1 mm crystals	[42]
**Molten salt (Ni catalyst)**	Bulk	1150 °C, slow cooling	Lower temperature, enables isotopically pure BP	Requires catalyst, slow cooling for large crystals	µm- to mm-scale crystals	[45]
**Solid-phase reaction ball-milling**	Nanoparticles	Ball-milling post-synthesis	High purity, avoids high-pressure setups	Requires post-processing for nanoparticles	Submicron particles (no yield data)	[29]
**Molten salt**	Nanoparticles	NaCl:KCl:LiCl, 1100 °C	Simplified process, high yield	Limited size control for ultrafine particles	74% yield	[46]
**CVD#1**	Nanowires	800–1000 °C	Tunable n-/p-type nanowires	Requires precise gas-phase control	Uniform nanowires (no yield)	[44]
**VLS growth**	Nanowires	1050 °C, Ni catalyst	High aspect ratio (10^4^), elastic microwires	Catalyst residue may affect applications	Length > 250 µm, diameter > 250 nm	[87]
**CVD#2**	Film	1000–1100 °C, low pressure	High crystallinity, preferred orientation	Lattice mismatch with Si/Sapphire	µm-thick films	[43]
**SiO_2_-assisted solid-state reaction**	Film	Annealing in sealed ampoule	Ultrathin nanosheets (~4 nm), water-dispersible	Lateral size inhomogeneity, limited thickness control	Sub-10 nm thickness	[49]
**Reactive sputtering**	Film	Annealing at 1000 °C	Amorphous-to-crystalline transition, doping control	High annealing temperature required	–	[40]

## Data Availability

Not applicable.

## Abbreviations

The following abbreviations are used in this manuscript:

BP	Boron phosphide
CVD	Chemical vapor deposition
*h*-BN	Hexagonal boron nitride
2D	Two-dimensional
*c*-BP	Cubic boron phosphide
*h*-BP	Hexagonal boron phosphide
TG-DTA	Thermal gravimetry/differential thermal analysis
TDTR	Time-domain thermoreflectance
*c*-BN	Cubic boron nitride
DFT	Density functional theory
BAs	Boron arsenide
PL	Photoluminescence
HCl	Hydrochloric acid
H_2_SO_4_	Sulfuric acid
HNO_3_	Nitric acid
NCs	Nanocrystals
BPSG	Borophosphosilicate glass
1D	One-dimensional
SEM	Scanning electron microscopy
TEM	Transmission electron microscopy
HRTEM	High-resolution transmission electron microscopy
EDX	Energy-dispersive X-ray
XRD	X-ray diffraction
VLS	Vapor–liquid–solid
VS	Vapor–solid
FETs	Field-effect transistors
PDMS	Polydimenthylsiloxane
PI	Polyimide
MBP	Milled boron phosphide
NFC	Nanofibrillated cellulose
BNNS	boron nitride nanosheets
3D	Three-dimensional
HER	Hydrogen evolution reactions
M-S	Mott–Schottky
AQE	Apparent quantum efficiency
CO_2_RR	CO_2_ reduction reaction
UV-Vis	Ultraviolet–visible
NISCs	Sodium-ion hybrid supercapacitors
MCMB	Mesoporous carbon microspheres
CDI	Capacitive deionization
VI	Uranium
